# Supplementary material for: R1-32-like public antibodies acquire tolerance to SARS-CoV-2 antigenic drift through somatic hypermutation
Source: PLoS Biol. 2026 Sep 15;24(9):e3003996. doi: 10.1371/journal.pbio.3003996 (PMC13667928; doi:10.1371/journal.pbio.3003996)

Fig.S4

The original WB images are outlined with blue boxes.

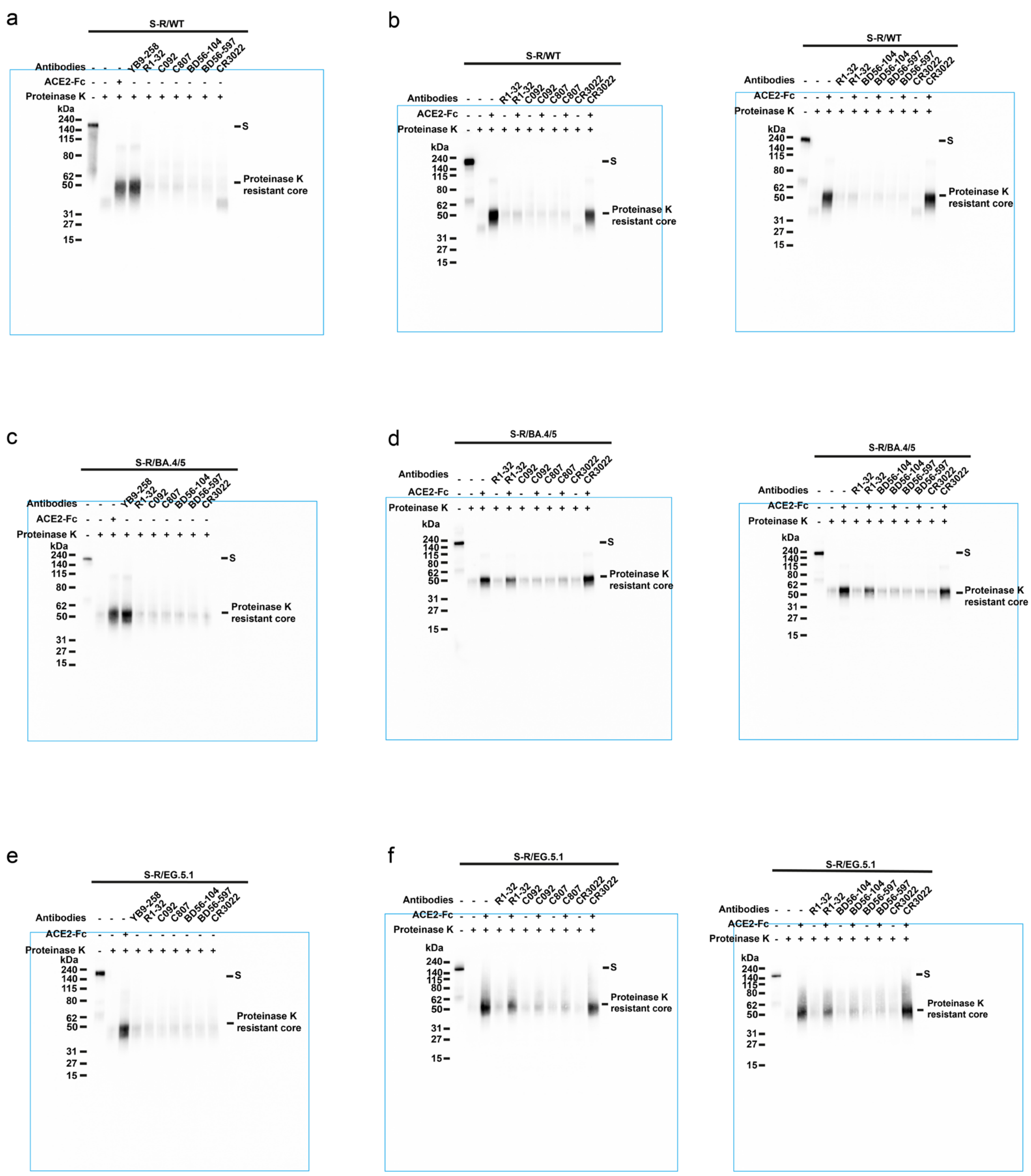

Supplement: Supplementary file 1 [file pbio.3003996.s043.pdf]
