# Supplementary material for: R1-32-like public antibodies acquire tolerance to SARS-CoV-2 antigenic drift through somatic hypermutation
Source: PLoS Biol. 2026 Sep 15;24(9):e3003996. doi: 10.1371/journal.pbio.3003996 (PMC13667928; doi:10.1371/journal.pbio.3003996)
Supplement: Supplementary file 3 [file pbio.3003996.s045.docx]

Response to Reviewers

We agree with the reviewers’ concerns and have revised the manuscript accordingly. We also performed all the requested experiments and incorporated the new results. We believe that these revisions and additional data have substantially strengthened the manuscript.

A summary of modifications to the manuscript is provided below:

1. Investigated the molecular basis of ZL525 glycan tolerance through additional experiments **(newly added Fig. S18)** and incorporated the findings into the Results and Discussion sections.
2. Functional validation of ZL525 HCDR2 grafting into other antibodies **(Fig. A in the response letter)**
3. Provided detailed descriptions of the AI model training dataset, data partitioning strategy, and similarity-based approaches to minimize data leakage **(revised Results and Methods)**.
4. Added AI model evaluation results **(revised Fig. 6b and newly added Fig. S16a and b)**.
5. Experimentally validated AI model predictions by testing top-ranked and low-ranked antibody candidates, demonstrating enrichment for broadly reactive antibodies among top-ranked candidates **(newly added Fig. S16c and d)**.
6. Additional structural and mechanistic interpretations: provided additional interpretation of spike conformational inhibition assays; explained the effects of R346 mutations on different antibodies; further clarified the roles of light-chain SHMs of C092 in maintaining antibody breadth; explained the impact of the Beta variant on R1-32 antibody neutralization; clarified the molecular basis of N354 glycan-mediated escape and the differential effects on C092, C807, BD56-104, BD56-597, and ZL525; corrected our use of “synergistic effect” to “additive effect”; and toned down the claims regarding the role of the AI model in antibody discovery.
7. Additional data summary and manuscript revisions: added a summary table compiling L452/F490 mutations across SARS-CoV-2 variants and the presence of the N354 glycosylation site; clarified ambiguous statements, incorporated appropriate references, and optimized figures and figure legends to improve manuscript clarity.

Reviewer #1: This study is comprehensive and content-rich, systematically elucidating how IGHV1-69 public antibodies acquire tolerance to SARS-CoV-2 antigenic drift through somatic hypermutation (SHM), and identifying the ultrapotent broadly neutralizing antibody ZL525 through AI-based discovery. However, further adjustments and improvements are necessary regarding the structural basis, functional validation, and reproducibility of the AI methodology.

1. Antibodies C092, C807, BD56-104, and BD56-597 acquire tolerance to the L452R and F490S mutations through SHM; however, they show varying degrees of sensitivity to the R346T mutation such as BQ.1.1. Is there structural evidence suggesting that the interaction modes of HCDR3 or LFR3 with R346 differ among these antibodies?

Response: We thank the reviewer for this insightful question. We agree with the reviewer that these antibodies exhibit varying degrees of sensitivity to the R346T_SARS2_ mutation, as exemplified by their differential neutralization of BQ.1.1. Notably, R346T_SARS2_ has been identified as a key immune escape mutation in multiple previous studies (DOI: 10.1016/j.cell.2022.12.018; DOI:10.1080/22221751.2024.2402880).

The differential sensitivity of these antibodies to the R346T_SARS2_ mutation is consistent with their distinct structural interactions with residue R346_SARS2_. Structural analyses revealed a conserved interaction pattern among C092, C807, BD56-104, and BD56-597, in which the backbone atom of HCDR2 residue Y60_H_ (or F60_H_) forms hydrogen bonds with R346_SARS2_. Because this interaction is mediated by the peptide backbone rather than the side chain, it is expected to be relatively insensitive to the R346T_SARS2_ substitution **(Fig. 3)**.

However, additional side-chain interactions with R346_SARS2_ are observed in C092 and BD56-104 **(Fig. 3a and c)**. In C092, residue D99_L_ in LCDR3 forms salt bridges with R346_SARS2_, whereas in BD56-104, HFR3 residue E62_H_ forms additional salt bridges with R346_SARS2_. Consequently, the R346T_SARS2_ substitution would disrupt these favorable electrostatic interactions, thereby providing a structural explanation for the relatively greater sensitivity of C092 and BD56-104 to the R346T-containing variant BQ.1.1 observed in neutralization assays.

We added a comment in the “Affinity maturation broadens the activity of R1-32-like antibodies toward SARSr-CoV RBDs” section (Line 439-442 in the new track-changes manuscript), when rationalizing testing the effect of R346T_SARS2_ on antibody binding.

“Of note, although R346T_SARS2_ lies at the periphery of the R1-32-like antibody epitope, it has been identified as a key immune-escape mutation in several previous studies (DOI: 10.1016/j.cell.2022.12.018; DOI: 10.1080/22221751.2024.2402880). The R346_SARS2_ side chain directly forms salt bridges with residues in C092 and BD56-104 (**Fig. 3a and c**).”

2. The mutant R1-32-AAM was engineered by introducing seven SHM sites into R1-32, yet it still lacks neutralizing activity against variants harboring the N354 glycosylation, such as KP.3. Does this imply that these SHM sites are insufficient to overcome the glycan barrier? Does ZL525 possess unique mutations beyond these seven sites?

Response: We thank the reviewer for this insightful question. Our results suggest that the seven SHMs introduced into R1-32-AAM are insufficient to overcome the glycan barrier imposed by the N354_SARS2_ glycosylation present in variants such as KP.3.

To date, ZL525 is the most mutation-tolerant R1-32-like public antibody identified in our study and retains robust activity against variants carrying the N354_SARS2_ glycosylation. Compared with R1-32-AAM, ZL525 contains two distinctive HCDR2 SHM-introduced residues, A55_H_ and M57_H_. Structural analysis indicates that SHM-introduced smaller or more flexible hydrophobic residues (L/F55A_H_ and I/T57M_H_) in HCDR2 likely confer enhanced conformational adaptability, allowing ZL525 to engage the altered hydrophobic surface formed by F490_SARS2_, L492_SARS2_, and the substituted L452W_SARS2_, while counteracting the shielding effect of the newly introduced N354_SARS2_ glycan **(Fig. 6f and Fig. S17a and b)**.

This structural hypothesis is further supported by our newly performed germline reversion experiments **(newly added Fig. S18)**. Simultaneous reversion of A55_H_ and M57_H_ to their germline residues in HCDR2 (L55_H_ and I57_H_) reduced the binding affinity of ZL525 to N354_SARS2_ glycosylated RBDs of BA.2.86, JN.1, KP.2, and KP.3 by approximately 3.2- to 10-fold **(newly added Fig. S18c and d)**, supporting their important contributions to maintaining affinity to RBDs containing the N354_SARS2_ glycan. However, this reverted ZL525 variant still retained higher binding affinity toward N354_SARS2_ glycosylated RBDs than the previously antibodies tolerant only to point mutations (C092, C807, BD56-104, and BD56-597), suggesting that additional SHMs or other sequence features contribute to the affinity of ZL525 towards N354_SARS2_ glycosylated RBDs **(Fig. S17d)**. Further germline reversion analyses revealed that additional SHMs, including HCDR1 P33_H_, HFR3 D59_H_, LCDR1 N26_L_, and LFR3 D70_L_, also contribute to the affinity of ZL525 towards N354_SARS2_ glycosylated RBDs **(newly added Fig. S18c and d)**. Together, these results identify A55_H_ and M57_H_ as the principal determinants of ZL525 binding to N354_SARS2_-glycosylated RBDs, while additional SHMs and sequence features distributed across both the heavy and light chains collectively enhance its exceptional glycan tolerance.

We have also incorporated this new result into the revised manuscript (Line 590-619 in the new track-changes manuscript):

“To define the molecular basis of ZL525 glycan tolerance, we systematically reverted its SHMs to their corresponding germline residues. Structural analysis identified A55_H_ and M57_H_ in HCDR2 as potentially important SHM-introduced residues involved in epitope recognition (**Fig. 6f**). Germline reversion of these two residues (ZL525-A55L_H_+M57I_H_) reduced binding affinity for N354_SARS2_-glycosylated RBDs by approximately 3.2- to 10-fold, demonstrating their substantial contribution to glycan tolerance (**Fig. S18**). Nevertheless, this revertant retained higher affinity for N354_SARS2_-glycosylated RBDs than the previously identified point-mutation-tolerant antibodies C092, C807, BD56-104, and BD56-597, suggesting that additional SHMs or other sequence features contribute to the exceptional glycan tolerance of ZL525 (**Fig. S17d**).

Consistent with this interpretation, additional reversion of the HCDR2 residues T58_H_ and HFR3 residue D59_H_ (ZL525-A55L_H_+M57I_H_+T58A_H_+D59N_H_) caused a further 1.1- to 2.6-fold reduction in binding affinity compared with reversion of the two HCDR2 residues alone. Simultaneous reversion of SHMs in HCDR1, HCDR2, and HFR3 (ZL525-A55L_H_+M57I_H_+T58A_H_+D59N_H_+H32Y_H_+P33A_H_) resulted in an additional 1.4- to 3.6-fold loss of affinity relative to the HCDR2 and HFR3 revertant. Structural inspection further identified four SHM-derived residues, A55_H_ and M57_H_ in HCDR2, D59_H_ in HFR3, and P33_H_ in HCDR1, that directly contribute to epitope recognition. Combined reversion of these four residues (ZL525-A55L_H_+M57I_H_+D59N_H_+P33A_H_) caused an affinity loss comparable to that observed upon complete reversion of the HCDR1, HCDR2, and HFR3 SHMs, indicating that these four residues account for most of the heavy-chain SHM-mediated effect (**Fig. S18**).

Additional reversion of LCDR SHMs in the background of the four-residue heavy-chain revertant further reduced binding affinity for the KP.2 and KP.3 RBDs by approximately 1.1- to 1.7-fold, indicating that light-chain SHMs also contribute to recognition of N354_SARS2_-glycosylated RBDs (**Fig. S18**). Together, these results identify A55_H_ and M57_H_ as the principal determinants of ZL525 binding to N354_SARS2_-glycosylated RBDs, while additional SHMs and sequence features distributed across both the heavy and light chains collectively enhance its exceptional glycan tolerance.”

3. In Fig 1 and Fig S4, R1-32 remained effective in the conformational change inhibition assay but completely lost its neutralizing activity. The authors should provide further clarification on this discrepancy.

Response: We sincerely thank the reviewer for this insightful comment. As shown in Fig. S4, although all five antibodies (R1-32, C092, C807, BD56-104, and BD56-597) effectively inhibited the transition of WT spike to the postfusion conformation, R1-32 exhibited a marked reduction in its inhibitory activity against BA.4/5 (containing L452R_SARS2_) and EG.5.1 (containing F490S_SARS2_) spikes compared with WT. This reduction is consistent with its decreased binding against variants carrying substitutions at these positions.

We believe that the apparent discrepancy arises from the distinct functional thresholds measured by the two assays. Although R1-32 is highly sensitive to substitutions at L452_SARS2_ and F490_SARS2_, these mutations do not completely abolish its binding to variant spike proteins, especially at high concentrations ([S-protein] =1 mg/ml (7.09 uM); [IgG] = 1.17 mg/ml (7.80 uM)) used for the ligand-induced conformational change assay **(Fig. S4)**. Indeed, R1-32 retains measurable binding activity against several variants carrying mutations at these positions, including Kappa, Delta, Lambda, BA.4/5, BF.7, BQ.1.1, and XBB **(Fig. 1c)**. Consequently, the residual binding of R1-32 to BA.4/5 and EG.5.1 spike proteins likely remains sufficient to partially inhibit their transition to the postfusion conformation, resulting in detectable inhibitory activity in the conformational transition assay under the conditions we used.

In contrast, pseudo- and authentic viral neutralization assays were performed at a substantially lower IgG concentration (highest [IgG] = 0.01 mg/mL (0.067 μM) for pseudoviral neutralization or highest [IgG] = 0.1 mg/mL (0.67 μM) for authentic viral neutralization). Moreover, effective neutralization likely requires a much greater degree of functional blockade, with most spike proteins on the virion needing to be inactivated or destroyed to prevent infection. Because productive viral entry may still be mediated by a small subset of functional spikes on the virion surface, partial inhibition of spike conformational transitions may be insufficient to block infection. Thus, although R1-32 retains residual binding and partially inhibits the conformational transition, these effects are insufficient to induce the degree of spike inactivation or destruction required for effective neutralization, resulting in the loss of detectable neutralizing activity against these variants.

We have provided further clarification on this discrepancy in the revised manuscript (Line 260-263) as follows:

“Furthermore, although R1-32 retains residual binding and partially inhibits ligand-induced spike conformational changes, its loss of detectable neutralizing activity against these variants suggests that the reduced binding and incomplete inhibition are insufficient to induce the extent of spike inactivation or disruption required to effectively block viral entry.”

4. Fig 3 shows that the LFR3 and LCDR3 regions of C092 are involved in RBD binding, whereas the other three antibodies do not exhibit similar characteristics. Can amino acid substitution experiments be conducted to validate the contribution of these LC regions to breadth?

Response: We sincerely appreciate the reviewer’s insightful suggestion. In fact, we have already performed germline reversion experiments targeting SHM-introduced residues in the light chain to assess the contribution of key light chain somatic hypermutations (SHMs) in C092, and these data are presented in **Fig. 4b**.

Specifically, based on our structural analysis, we selected three key light-chain residues involved in RBD recognition, namely LCDR1 residue N26_L_, LFR3 residue D70_L_, and LCDR3 residue D99_L_, and assessed their contributions to antibody breadth through germline reversion analysis. Our results showed that reversion of these residues to their germline counterparts substantially reduced the binding affinity of C092 to the RBDs of all tested variants, with reductions typically ranging from ~10- to 100-fold **(Fig. 4b)**. These findings demonstrate that light chain residues, including those located in LFR3 and LCDR3, make important contributions to the breadth of C092.

We have further clarified this point in the revised manuscript (Line 333-336) to better emphasize the functional roles of these light chain residues as follows:

“Likewise, reverting the light chain SHM-introduced residues (N26S_L_, D70G_L_, and D99G_L_) to germline reduced binding to the same set of RBDs by ~10-100-fold, demonstrating that light chain residues make important contributions to the breadth of C092 (**Fig. 4b and Fig. S11a**).”

5. Did the AI model account for non-affinity factors, such as glycan shielding? Is ZL525's activity against KP.3 a serendipitous discovery?

Response: We thank the reviewer for this important question. The AI model used in this study was primarily designed to identify antibodies with broad binding potential based only on sequence and neutralization data (which is largely a surrogate of affinity). It did not explicitly incorporate non-affinity factors, such as glycan shielding, as input parameters during model training or candidate selection.

Therefore, the potent activity of ZL525 against KP.3 and other variants carrying the N354_SARS2_-glycosylation was not an explicitly optimized feature of the AI model. Rather, it was identified during the experimental characterization of AI-prioritized candidates.

Importantly, we do not consider this observation to be entirely serendipitous. Our AI model was likely biased toward identifying antibodies with high predicted affinity, and ZL525 ranked among the top-scoring candidates. This prediction was subsequently validated experimentally, as ZL525 exhibited some of the highest binding affinities across a broad panel of SARS-CoV-2 variants and sarbecoviruses **(Figs. 5a and 6e)**. The extensive affinity maturation of ZL525 may also have contributed to its substantially broadened antigen recognition. Consistent with this possibility, our subsequent structural and functional analyses showed that the maturation process conferred an unexpected but advantageous capacity to tolerate glycan-mediated antigenic changes. Thus, the glycan tolerance of ZL525 may represent an emergent consequence of extensive affinity maturation that simultaneously optimized binding affinity and breadth of antigen recognition.

6. Does the glycan tolerance of ZL525 originate from the conformational plasticity of HCDR2? It may be beneficial to graft HCDR2 of ZL525 onto the frameworks of the four antibodies, such as C092, to verify whether this confers glycan tolerance.

Response: We thank the reviewer for this good suggestion. To investigate whether the glycan tolerance of ZL525 originates from the conformational plasticity of HCDR2, we newly performed additional experiments to comprehensively assess the effects of germline reversion of key somatic hypermutations in ZL525 **(newly added Fig. S18)**. We have also incorporated this new result into the revised manuscript (Line 590-619). Please also see our reply to your major point 2.

These findings support the notion that the conformational adaptability conferred by HCDR2 is an important determinant of glycan tolerance in ZL525. However, our structural and mutational analyses further indicate that HCDR2 alone is not sufficient to fully explain the exceptional glycan tolerance of ZL525. Additional SHMs located in HCDR1, HFR3, and the light chain also contribute to ZL525’s affinity towards N354_SARS2_-glycosylated RBDs **(newly added Fig. S18)**. We have clarified these points in the revised manuscript and now emphasize that HCDR2 is an important, but not exclusive, determinant of glycan tolerance in ZL525.

Importantly, in response to the reviewer's suggestion, we newly introduced the key SHMs in HCDR2 and surrounding HFR3 (A55_H_, M57_H_, T58_H_, and D59_H_) identified in ZL525 into the other four antibodies (C092, C807, BD56-104, and BD56-597) examined in this study. Regrettably, these substitutions resulted in only modest improvements in glycan tolerance **(as shown in Fig. A)**. This observation suggests that the functional effects of SHMs are strongly dependent on antibody sequence context. Accordingly, the contribution of any individual SHM is likely to be influenced by the presence of other SHM-introduced residues or other sequence features. Optimal glycan tolerance may therefore arise from the combined effects of multiple factors rather than from the introduction of any single mutation or mutations in a single CDR region. Because introducing the HCDR2 and adjacent HFR3 SHMs (A55_H_, M57_H_, T58_H_, and D59_H_) into the other four antibodies, C092, C807, BD56-104, and BD56-597, resulted in only modest improvements in binding to RBDs bearing the N354_SARS2_ glycan, we did not include these results in the revised manuscript.

**Fig. A. Binding affinities of engineered C092, C807, BD56-104, and BD56-597 variants bearing the ZL525 HCDR2 graft to RBDs.**

Binding curves of C092, C807, BD56-104, and BD56-597 and their mutants to the wild-type RBD and the glycosylated RBDs from BA.2.86, JN.1, KP.2, and KP.3. Affinity changes of the antibody mutants were normalized to the *K*_D_ values of the corresponding wild-type antibodies (set as 1%).

Reviewer #2: Here is an assessment of the Niu et al manuscript, focusing primarily on evaluation of the AI model aspects:

The model is described as a sequence-embedding-based affinity predictor. Independent RoFormer language models are used for antibody heavy and light chain embeddings, ESM2 is used for antigen embeddings, and an MLP is fit on top of the embeddings to predict antibody-antigen binding affinity.

1. One challenge is that the description of the model training data is inconsistent and insufficiently specific to allow for close review. The abstract says the model was trained on integrated structural, neutralization, and binding data, whereas the Methods section says it was fine-tuned on a consolidated affinity dataset assembled from multiple public sources. Those descriptions are not equivalent, and leave it unclear what training data was actually used for which model components.

Response: We thank the reviewer for this comment and apologize for the confusion caused by our previous unclear description. We have now clarified the description of the training data throughout the manuscript to enhance clarity regarding the training data utilized by the model. We curated a total of 752 IC_50_-labeled neutralization data (which is largely a surrogate of affinity) of various SARS-CoV-2 antibodies neutralizing WT SARS-CoV-2 virus entries from multiple publicly available sources to train the model. These neutralization data are assembled from the following reports: doi:10.1038/s41586-022-05644-7; doi:10.1016/j.immuni.2021.07.008; doi:10.1038/s41586-022-04980-y; doi:10.1038/s41586-021-03207-w; doi:10.1038/s41586-021-03696-9; doi:10.1038/s41586-021-04385-3; doi:10.1084/jem.20221006; doi:10.1038/s41467-023-35927-0; doi:10.1126/sciimmunol.abq3511.

The corresponding statements in the **Abstract** (Line 46-50 in the new track-changes manuscript) and **Methods** (Line 1017-1025) sections have been revised as follows:

“Using an AI model trained on extensive neutralization data, we further identified ZL525, an ultrapotent R1-32-like antibody with pan-SARS-CoV-2 variant activity, including against the highly evasive KP.3 variant carrying the N354_SARS2_ glycosylation, and broad sarbecovirus cross-reactivity extending to SARS-CoV-1.”

“The model was fine-tuned on a consolidated IC_50_-labeled neutralization dataset assembled from multiple public sources (doi:10.1038/s41586-022-05644-7; doi:10.1016/j.immuni.2021.07.008; doi:10.1038/s41586-022-04980-y; doi:10.1038/s41586-021-03207-w; doi:10.1038/s41586-021-03696-9; doi:10.1038/s41586-021-04385-3; doi:10.1084/jem.20221006; doi:10.1038/s41467-023-35927-0; doi:10.1126/sciimmunol.abq3511). A total of 752 data entries regarding the neutralizing activity of antibodies against wild-type SARS-CoV-2 were collected. The entire dataset was split into training, validation, and test sets with a ratio of 80%, 10%, and 10%, respectively, while strictly ensuring that the sequence similarity of antibody HCDR3 regions between any sample in the test set and those in the training and validation sets remained below 70%. Here, similarity was computed based on the HCDR3 regions of the antibody sequences using MMseqs2.”

2. The manuscript itself does not adequately report model evaluation. The authors don't present a train/validation/test split, a data-leak avoidance strategy (e.g. a sequence-similarity-based exclusion criterion across train/validation/test splits), an external validation set, calibration analysis, or performance metrics demonstrating the model predicts affinity well.

Response: We thank the reviewer for this comment. We have incorporated a detailed description of the dataset partitioning strategies into the manuscript, along with a rigorous similarity-based strategy to mitigate data leakage. Additionally, comprehensive performance evaluations of the proposed model and baseline methods on the test sets have been added to the **Results** section.

A total of 752 neutralization data points were split into training, validation, and test sets with a ratio of 80%, 10%, and 10%, respectively, while strictly ensuring that the sequence similarity of antibody HCDR3 regions between any sample in the test set and those in the training and validation sets remained below 70%.

We evaluated our model against competing approaches, PIPR (doi.org/10.1093/bioinformatics/btz328) and one-hot based baseline, using multiple metrics, including Pearson’s correlation coefficient, Spearman’s correlation coefficient, RMSE (Root Mean Squared Error), and MAE (Mean Absolute Error). The proposed model achieved Pearson’s and Spearman’s correlation coefficients of 0.6266 and 0.5969, with RMSE and MAE values of 0.7394 and 0.5634, respectively **(revised Fig. 6b and newly added S16a)**. The sequence-based method PIPR showed lower performance compared to our approach, yielding Pearson’s correlation, Spearman’s correlation, RMSE, and MAE values of 0.4655, 0.4456, 0.8333, and 0.6674, respectively **(revised Fig. 6b and** **newly added S16a)**. These results demonstrate the effectiveness of our proposed method. Furthermore, we established a baseline model by replacing the language model features with one-hot encoded vectors. Compared with language model-based methods, the baseline model exhibited a 40.81% decrease in Pearson’s correlation (0.3709) and a 34.33% decrease in Spearman’s correlation (0.3920), further demonstrating the critical role of language model features **(revised Fig. 6b and newly added S16a)**. We have added the detailed data partitioning and performance metric analysis to the **Results** section of the manuscript as follows (Line 501-524):

“This deep learning model was fine-tuned using 752 experimentally determined neutralization measurements of antibodies against wild-type SARS-CoV-2. The dataset was partitioned into training, validation, and test sets at a ratio of 80%, 10%, and 10% using a stringent similarity-based splitting strategy. Antibody HCDR3 sequence similarity between each test-set sample and all samples in the training and validation sets was maintained below 70%.”

“We evaluated our model against competing approaches, PIPR (doi.org/10.1093/bioinformatics/btz328) and one-hot based baseline, using multiple metrics, including Pearson’s correlation coefficient, Spearman’s correlation coefficient **(Fig. 6b)**, RMSE (Root Mean Squared Error), and MAE (Mean Absolute Error) **(Fig. S16a)**. The proposed model achieved Pearson’s and Spearman’s correlation coefficients of 0.6266 and 0.5969, with RMSE and MAE values of 0.7394 and 0.5634, respectively. In contrast, the sequence-based method PIPR obtained corresponding Pearson’s correlation, Spearman’s correlation, RMSE, and MAE values of 0.4655, 0.4456, 0.8333, and 0.6674. These results demonstrate the effectiveness of our proposed method. Furthermore, we established a baseline model by replacing the language model features with one-hot encoded vectors. Compared with language model-based methods, the baseline model showed a 40.81% decrease in Pearson’s correlation and a 34.33% decrease in Spearman’s correlation, further demonstrating the critical contribution of the learned language-model features.”

3. The manuscript says that top-scoring antibodies were prioritized for experimental validation, but it is not clear how many ranked candidates were tested. The paper mainly showcases a single successful lead, ZL525, which was predicted to have higher affinity and was then experimentally validated.

Response: We thank the reviewer for this important comment. In this study, a total of 190 antibody candidates were ranked by the AI model **(newly added Fig. S16b)**. For experimental validation, we selected five top-ranked antibodies, including ZL525, and nine low-ranked antibodies for functional testing **(****newly added Fig. S16c and d)**. Among the five top-ranked antibodies, four exhibited broad reactivity against multiple SARS-CoV-2 variants, whereas only one (T45) showed limited breadth of reactivity. In contrast, only one (T49) of the nine low-ranked antibodies exhibited broad reactivity, indicating a clear enrichment of broadly reactive antibodies among the top-ranked group **(newly added Fig. S16c and d)**.

Importantly, ZL525 displayed the strongest and most consistent broad reactivity among all tested antibodies. It showed high binding activity against multiple SARS-CoV-2 variants, including WT, Delta, Lambda, BQ.1.1, EG.5.1, JN.1, and KP.3 **(newly added Fig. S16c and d)**. These results further support the effectiveness of our AI-based prioritization strategy.

We have also incorporated this new result into the revised manuscript (Line 526-541):

“The affinity prediction model was subsequently applied to a curated panel of 190 R1-32-like antibodies **(Fig. S16b)**. Notably, the AI model assigned the highest affinity score to ZL525, whereas the antibodies R1-32 and FC08 ranked among the lowest-scoring candidates. To experimentally evaluate the predictive performance of the model, we selected the top five AI-ranked antibodies, including ZL525, together with nine low-ranked antibodies for functional validation. Among the five top-ranked antibodies, four exhibited broad neutralizing activity, whereas only one (T45) showed limited breadth. In contrast, only one of the nine low-ranked antibodies (T49) displayed broad neutralization, while the remaining antibodies exhibited limited activity against antigenically divergent variants **(Fig. S16c and d)**. These results demonstrate that the AI model effectively enriched for broadly neutralizing antibodies and accurately prioritized candidates with improved breadth. Among all candidates, ZL525 displayed the highest predicted score together with the broadest neutralizing profile and was therefore selected for further characterization.”

4. Most importantly, there is no negative-control validation, for example with experiments selecting low-ranked or random candidates to test whether the model actually enriched for hits. Without that, and especially combined with the lack of reporting on model evaluation/performance, the manuscript does not provide evidence to demonstrate the value is added by the affinity prediction model.

Response: We thank the reviewer for this important comment. We agree that appropriate control analyses are essential for evaluating model performance.

To address the concern regarding negative-control validation, we tested both top-ranked and low-ranked antibody candidates selected from the 190 AI-ranked antibodies. Specifically, we selected five top-ranked antibodies and nine low-ranked antibodies for experimental testing. Among the five top-ranked antibodies, four exhibited broad reactivity against multiple SARS-CoV-2 variants **(newly added Fig. S16c and d)**. In contrast, only one of the nine low-ranked antibodies showed similar broad reactivity **(newly added Fig. S16c and d)**, indicating that the top-ranked group is significantly enriched for broadly reactive antibodies compared with the low-ranked group.

These results demonstrate that the AI-based ranking strategy effectively enriches for antibodies with favorable breadth-related properties, thereby supporting the added value of the affinity prediction model. We have also incorporated this new result into the revised manuscript (Line 526-541).

5. In its current form, the structural and antibody-engineering parts of the manuscript are strong but the ML elements are weak and will need convincing dataset composition, split strategy, held-out-set performance, number of experimentally tested ranked candidates, and, if possible, prospective validation against negatives or a null model.

Response: We thank the reviewer for the constructive summary. We have carefully addressed the reviewer’s concerns in our responses to the preceding four comments, and the corresponding revisions have been incorporated into the revised manuscript. Specifically, we have now provided dataset composition, split strategy, held-out-set performance, and experimental validation that included both top-ranked and low-ranked candidate antibodies to serve as a negative control. These additions collectively demonstrate the predictive value of our AI model and its effective enrichment of broadly neutralizing antibodies.

Reviewer #3: In this study, Niu et al. characterized how a class of IGHV1-69 public antibodies targeting SARS-CoV-2 RBD overcomes escape mutations at L452 and F490 that are found in multiple SARS-CoV-2 variants via the acquisition of somatic hypermutations. Key somatic hypermutations were identified through an in-depth characterization of four members in this public antibody class with broad reactivity (C092, C807, BD56-104, and BD56-597). These somatic hypermutations also improve cross-reactivity breadth against other SARSr-CoV strains. These results provide a mechanistic understanding of how affinity maturation drives the development of cross-reactivity breadth for this class of public antibodies. At the end, an AI model was trained to identify the antibody ZL525, which belongs to the same public antibody class as C092, C807, BD56-104, and BD56-597, but with even broader cross-reactivity. Overall, this study is well-executed and provides important insights into the interplay between SARS-CoV-2 antibody responses and viral evolution. However, there are several concerns that need to be addressed.

Major comments:

1. According to lines 150-151, Beta has no mutations at positions 452 and 490. However, the binding and neutralization of germline-like R1-32 against Beta is much weaker than against the WT (Figure 1c, e, f, and g). Is there a structural explanation?

Response: We thank the reviewer for this comment. Although the Beta variant does not contain mutations at positions L452_SARS2_ or F490_SARS2_, it harbors the E484K_SARS2_ substitution, which lies within the R1-32 epitope, as previously reported in our earlier study (doi.org:10.1038/s41564-022-01235-4; **Extended Data Fig. 5 and Supplementary Fig. 1**). Therefore, the reduced binding and neutralization activity of germline-like R1-32 against the Beta variant may be attributed, at least in part, to the presence of the E484K_SARS2_ mutation.

Given that residue E484_SARS2_ lies within the R1-32 antibody footprint, the E484K_SARS2_ substitution may alter local electrostatic interactions and perturb the conformational landscape of the epitope, thereby affecting antibody recognition. Beyond the above possibility, our current understanding of R1-32-like antibodies suggests that their neutralizing activity may also be modulated by spike stability and conformational dynamics, which could influence their ability to induce spike disruption. The Beta-associated substitutions K417N_SARS2_ and N501Y_SARS2_ have been reported to alter spike conformational properties, including the RBD up-down ratios and receptor-binding activity (DOI: 10.1038/s41467-022-28768-w; DOI: 10.1126/science.abi6226). We therefore cannot exclude the possibility that these effects also contribute to the reduced neutralization sensitivity of R1-32-like antibodies.

2. The observation described in lines 367-368: "combining HCDR1 germline reversion with the HCDR3 Y109A_H_ substitution in C092, C807, and BD56-597 produced an additive reduction in binding" contradicts with the claim in lines 369-370: "distinct antigen-contact regions act synergistically to achieve high-affinity binding." By definition, additive means no synergy.

Response: We thank the reviewer for this important comment. We agree that the observed effects do not provide clear evidence of synergy among the different CDR regions. Rather, our data support an additive model in which multiple antigen-contacting regions additively contribute to high-affinity binding. We have revised the manuscript accordingly (Line 375-378 in the new track-changes manuscript). The revised text now reads:

“Moreover, combining HCDR1 germline reversion with the HCDR3 Y109A_H_ substitution in C092, C807, and BD56-597 produced an additive reduction in binding **(Fig. 4b-e and Fig. S11)**, indicating that distinct antigen-contact regions jointly contribute to high-affinity binding in a combinatorial manner.”

3. Lines 535-540: "In contrast, the AI-identified antibody ZL525 exhibits greater tolerance to this glycan-mediated shielding, likely due to structural adaptations introduced by somatic hypermutation… and highlight ZL525 as a broadly neutralizing antibody capable of overcoming this glycan-mediated escape mechanism." This is a very interesting point. To increase impact of this study, the authors should elaborate on the proposed mechanism by analyzing how N354 glycan would affect the binding of C092, C807, BD56-104, and BD56-597, but not ZL525.

Response: We thank the reviewer for this suggestion. From the cryo-EM structure of the ZL525:LP.8.1 RBD complex (containing the N354_SARS2_ glycan), we observed that the N354_SARS2_ glycosylation site lies within the epitope region recognized by R1-32-like public antibodies **(Fig. 6f, middle panel and Fig. S17a and b)**, suggesting that this glycan likely introduces steric hindrance and thereby affects the binding of C092, C807, BD56-104, and BD56-597.

Based on structural analysis together with germline reversion experiments, we propose that the impact of the N354_SARS2_ glycan on ZL525 binding may be explained by multiple, potentially complementary factors. In brief:

First, ZL525 contains the HCDR2 SHMs A55_H_ and M57_H_, which introduce smaller or more conformationally flexible hydrophobic residues that likely confer enhanced conformational adaptability. This increased adaptability may enable ZL525 to accommodate the remodeled hydrophobic surface formed by F490_SARS2_, L492_SARS2_, and L452W_SARS2_ substitution, while partially alleviating the steric shielding imposed by the newly introduced N354_SARS2_ glycan **(Fig. 6f, middle panel and Fig. S17a and b)**. Consistent with this hypothesis, reversion of A55_H_ and M57_H_ to their germline counterparts (L55_H_ and I57_H_) greatly reduced the binding affinity of ZL525 to glycosylated RBDs from BA.2.86, JN.1, KP.2, and KP.3 by approximately 3.2- to 10-fold **(****newly added Fig. S18c and d)**.

Second, the overall somatic hypermutation pattern of ZL525 may contribute to maintaining high affinity across diverse antigenic variants, thereby reducing the functional impact of glycan-mediated epitope shielding. Our germline reversion analyses further demonstrate that additional SHMs, including HCDR1 P33_H_, HFR3 D59_H_, LCDR1 N26_L_, and LFR3 D70_L_, also make substantial contributions to glycan tolerance **(****newly added Fig. S18c and d)**. Together, these findings indicate that the exceptional glycan tolerance of ZL525 arises from the combined effects of multiple SHMs.

We have included the above points in the final section of the **Results** section of the revised manuscript as the following results (Line 590-619):

“To define the molecular basis of ZL525 glycan tolerance, we systematically reverted its SHMs to their corresponding germline residues. Structural analysis identified A55_H_ and M57_H_ in HCDR2 as potentially important SHM-introduced residues involved in epitope recognition (**Fig. 6f**). Germline reversion of these two residues (ZL525-A55L_H_+M57I_H_) reduced binding affinity for N354_SARS2_-glycosylated RBDs by approximately 3.2- to 10-fold, demonstrating their substantial contribution to glycan tolerance (**Fig. S18**). Nevertheless, this revertant retained higher affinity for N354_SARS2_-glycosylated RBDs than the previously identified point-mutation-tolerant antibodies C092, C807, BD56-104, and BD56-597, suggesting that additional SHMs or other sequence features contribute to the exceptional glycan tolerance of ZL525 (**Fig. S17d**).

Consistent with this interpretation, additional reversion of the HCDR2 residues T58_H_ and HFR3 residue D59_H_ (ZL525-A55L_H_+M57I_H_+T58A_H_+D59N_H_) caused a further 1.1- to 2.6-fold reduction in binding affinity compared with reversion of the two HCDR2 residues alone. Simultaneous reversion of SHMs in HCDR1, HCDR2, and HFR3 (ZL525-A55L_H_+M57I_H_+T58A_H_+D59N_H_+H32Y_H_+P33A_H_) resulted in an additional 1.4- to 3.6-fold loss of affinity relative to the HCDR2 and HFR3 revertant. Structural inspection further identified four SHM-derived residues, A55_H_ and M57_H_ in HCDR2, D59_H_ in HFR3, and P33_H_ in HCDR1, that directly contribute to epitope recognition. Combined reversion of these four residues (ZL525-A55L_H_+M57I_H_+D59N_H_+P33A_H_) caused an affinity loss comparable to that observed upon complete reversion of the HCDR1, HCDR2, and HFR3 SHMs, indicating that these four residues account for most of the heavy-chain SHM-mediated effect (**Fig. S18**).

Additional reversion of LCDR SHMs in the background of the four-residue heavy-chain revertant further reduced binding affinity for the KP.2 and KP.3 RBDs by approximately 1.1- to 1.7-fold, indicating that light-chain SHMs also contribute to recognition of N354_SARS2_-glycosylated RBDs (**Fig. S18**). Together, these results identify A55_H_ and M57_H_ as the principal determinants of ZL525 binding to N354_SARS2_-glycosylated RBDs, while additional SHMs and sequence features distributed across both the heavy and light chains collectively enhance its exceptional glycan tolerance.”

In addition, we have expanded the **Discussion** section to further elaborate on the potential mechanism underlying why the N354_SARS2_ glycosylation impairs the binding of C092, C807, BD56-104, and BD56-597, while having minimal effects on ZL525 binding. The revised text now reads (Line 685-693):

“Structural analysis indicates that SHM-introduced smaller or more flexible hydrophobic residues (L/F55A_H_ and I/T57M_H_) in HCDR2 likely confer enhanced conformational adaptability, allowing ZL525 to engage the altered hydrophobic surface formed by F490_SARS2_, L492_SARS2_, and more importantly the substituted L452W_SARS2_ **(Fig. 6f)**, while counteracting the shielding effect of the newly introduced N354_SARS2_ glycan. In addition, SHMs distributed across both heavy and light chains collectively contribute to the glycan tolerance of ZL525. The combined effects of these SHMs may explain why N354_SARS2_ glycosylation markedly disrupts the binding of C092, C807, BD56-104, and BD56-597, but has only a minimal impact on ZL525 binding.”

4. Lines 595-598: "We have shown that the synergistic effect of sequence variation and glycan-mediated structural occlusion enables viral escape from previously identified mutation-tolerant R1-32-like antibodies (C092, C807, BD56-104, and BD56-597)." This claim of synergistic effect is not supported by any data in this study.

Response: We thank the reviewer for this important comment. We agree that our data do not directly demonstrate synergy between sequence variation and glycan-mediated structural occlusion. Our intention was to emphasize that both sequence variation and glycan-mediated shielding contribute to viral escape from previously identified mutation-tolerant R1-32-like antibodies. We have therefore revised the relevant statement in the manuscript to remove the implication of a “synergistic effect” and avoid overinterpretation.

The revised text now reads (Line 679-682):

“We have shown that both sequence variation and glycan-mediated structural occlusion contribute to viral escape from previously identified mutation-tolerant R1-32-like antibodies (C092, C807, BD56-104, and BD56-597).”

5. Lines 616-618: "This approach is particularly powerful for targeting rapidly evolving pathogens, like SARS-CoV-2, where conventional antibody discovery methods struggle to keep pace with viral escape variants." This claim needs to be toned down. Since the model requires training data from antibodies discovered via conventional methods against antigenic variants (lines 911-914), it remains inherently limited by the same constraints as conventional antibody discovery methods.

Response: We thank the reviewer for this important comment. We agree that the original wording may have overstated the advantages of our approach. As the reviewer correctly pointed out, the model relies on training data derived from antibodies identified through conventional discovery pipelines against antigenic variants and therefore shares inherent limitations associated with these underlying datasets. We did not intend to imply that AI-based approaches can replace conventional antibody discovery methods. Rather, we believe that such approaches can complement and accelerate conventional pipelines by prioritizing promising candidates and facilitating the identification of broadly reactive antibodies from large sequence repertoires.

In response, we have revised the manuscript by toning down the statement and removing the overclaim. The revised sentence now reads (Line 704-709):

“This approach may complement conventional antibody discovery strategies for rapidly evolving pathogens, such as SARS-CoV-2, by enabling more efficient identification of antibody candidates with broad reactivity against emerging viral variants.”

Minor comments:

1. Lines 85-86: "We have also previously identified a class of R1-32-like public antibodies elicited in more than 50% of COVID-19 convalescents early in the pandemic." A reference is needed.

Response: We sincerely thank the reviewer for pointing this out. We have now added the appropriate reference at this position in the revised manuscript to support this statement (Line 87).

2. Lines 96-98: "Intriguingly, we found that several members of the R1-32-like public antibody class, including C092, C807, BD56-104, and BD56-597, retain robust binding to S-proteins of viral variants carrying mutations at positions 452_SARS2_ and 490_SARS2_." A reference is needed.

Response: We sincerely thank the reviewer for pointing this out. We have now added the appropriate references in the revised manuscript to accurately cite the original studies describing C092, C807, BD56-104, and BD56-597 (Line 98).

3. Line 115: should "IGHV1-69 encoded" be "IGHV1-69/IGLV1-40 encoded" according to line 87? Relatedly, since this study identified convergent somatic hypermutations in the light chain, it does not make sense if those antibodies are encoded by different light chain V genes.

Response: We thank the reviewer for this important comment. We agree that the description at Line 115 was incomplete and may cause confusion. The antibodies discussed in this study are indeed encoded by IGHV1-69 paired with IGLV1-40, and thus share a common light chain V gene usage. We have clarified this point and updated the manuscript to consistently describe these antibodies as IGHV1-69/IGLV1-40 encoded (Line 116).

4. It will be helpful to include a table in the supplemental information to summarize the amino acid sequences at position 452 and 490 across different SARS-CoV-2 variants.

Response: We sincerely thank the reviewer for this helpful suggestion. In response, we have included a new summary table **(newly added Table S12)** in the Supplementary Information, summarizing the mutation profiles at positions 452 and 490 across different SARS-CoV-2 variants, as well as the presence or absence of the N354 glycosylation site.

5. Lines 186-187: "IC_50_ >10 µg/mL" Should that be ">100 µg/mL" according to Figure 1g?

Response: The IC_50_ value reported in Lines 186–187 refers to the pseudovirus neutralization assay shown in **Fig. 1e** rather than **Fig. 1g** (Authentic virus neutralization with 100 µg/mL as the highest IgG concentration used). In the pseudovirus neutralization assay, the highest antibody concentration tested was 10 µg/mL. Therefore, samples that failed to achieve 50% neutralization at this concentration were reported as having an IC_50_ >10 µg/mL. We have clarified this point in the revised manuscript to avoid any confusion.

We have revised the corresponding statement in the manuscript as follows (Line 188-189): “By contrast, germline-like R1-32 lost neutralization (IC_50_ >10 µg/mL) against Delta, Lambda, BA.4/5, BQ.1.1, and EG.5 pseudoviruses **(Fig. 1e)**.”

6. Figure 2: please clarify the meaning of "S-R/6P" in the figure legend.

Response: We thank the reviewer for pointing this out. We have revised the figure legend in the revised manuscript to include the following info (Line 1310-1312):

(S-R/6P: furin cleavage site (PRRA) deleted while retaining R685_SARS2_ at the S1/S2 site and stabilized by six proline (P) substitutions, also see Methods).

This also directs the readers to refer to detailed information regarding the construction of the S-R/6P spike protein provided in the Methods section.

7. Figure S4: for some of the panels, the label at the top of the figures do not align with the corresponding lane in the gel image.

Response: We sincerely thank the reviewer for pointing out this issue. We have carefully corrected the misaligned labels in **revised Figure S4** and replaced the figure in the revised manuscript accordingly.

8. Lines 487-488: "based on a consolidated antigen binding affinity dataset." What dataset is that? If it is from a previous study, please provide the reference.

Response: We sincerely thank the reviewer for this helpful comment. Following the reviewer's suggestion, we have revised this section and moved the detailed description of the dataset to the **Methods** section. Specifically, the dataset used for model fine-tuning was assembled from previously published studies, and all relevant references have now been explicitly cited in the revised manuscript. We have also clarified this point in the **Methods** section to provide a more comprehensive description of the data sources used for model training.

We have revised the corresponding statement in the manuscript as follows (Line 1017-1018):

The model was fine-tuned on a consolidated IC_50_-labeled neutralization dataset assembled from multiple public sources (doi:10.1038/s41586-022-05644-7; doi:10.1016/j.immuni.2021.07.008; doi:10.1038/s41586-022-04980-y; doi:10.1038/s41586-021-03207-w; doi:10.1038/s41586-021-03696-9; doi:10.1038/s41586-021-04385-3; doi:10.1084/jem.20221006; doi:10.1038/s41467-023-35927-0; doi:10.1126/sciimmunol.abq3511).

9. Line 533: "All four antibodies". Please indicate which four antibodies to avoid confusion.

Response: We sincerely thank the reviewer for pointing this out. To avoid confusion, we have revised the text to explicitly specify that the four antibodies referred to are C092, C807, BD56-104, and BD56-597.

We have revised the corresponding statement in the manuscript as follows (Line 583-586):

“All four antibodies (C092, C807, BD56-104, and BD56-597) exhibited restored high-affinity binding to these glycan-revertants, with markedly improved association rates (*k*_on_) **(Fig. S17d)**, confirming that the N354_SARS2_ glycosylation contributes to their escape.”
