# Supplementary material for: R1-32-like public antibodies acquire tolerance to SARS-CoV-2 antigenic drift through somatic hypermutation
Source: PLoS Biol. 2026 Sep 15;24(9):e3003996. doi: 10.1371/journal.pbio.3003996 (PMC13667928; doi:10.1371/journal.pbio.3003996)
Supplement: Peer review history

# Editor report: Decision Letter 0

**DOI:** 10.1371/journal.pbio.3003996.r001

**Authors:** Richard Hodge

21 Mar 2026

Dear Dr Xiong,

Thank you for submitting your manuscript entitled "Structural determinants of IGHV1-69 public antibodies conferring resilience to SARS-CoV-2 antigenic escape" for consideration as a Research Article by PLOS Biology.

Your manuscript has now been evaluated by the PLOS Biology editorial staff, as well as by an academic editor with relevant expertise, and I am writing to let you know that we would like to send your submission out for external peer review.

However, before we can send your manuscript to reviewers, we need you to complete your submission by providing the metadata that is required for full assessment. To this end, please login to Editorial Manager where you will find the paper in the 'Submissions Needing Revisions' folder on your homepage. Please click 'Revise Submission' from the Action Links and complete all additional questions in the submission questionnaire.

Once your full submission is complete, your paper will undergo a series of checks in preparation for peer review. After your manuscript has passed the checks it will be sent out for review. To provide the metadata for your submission, please Login to Editorial Manager (https://www.editorialmanager.com/pbiology) within two working days, i.e. by Mar 23 2026 11:59PM.

If your manuscript has been previously peer-reviewed at another journal, PLOS Biology is willing to work with those reviews in order to avoid re-starting the process. Submission of the previous reviews is entirely optional and our ability to use them effectively will depend on the willingness of the previous journal to confirm the content of the reports and share the reviewer identities. Please note that we reserve the right to invite additional reviewers if we consider that additional/independent reviewers are needed, although we aim to avoid this as far as possible. In our experience, working with previous reviews does save time.

If you would like us to consider previous reviewer reports, please edit your cover letter to let us know and include the name of the journal where the work was previously considered and the manuscript ID it was given. In addition, please upload a response to the reviews as a 'Prior Peer Review' file type, which should include the reports in full and a point-by-point reply detailing how you have or plan to address the reviewers' concerns.

During the process of completing your manuscript submission, you will be invited to opt-in to posting your pre-review manuscript as a bioRxiv preprint. Visit http://journals.plos.org/plosbiology/s/preprints for full details. If you consent to posting your current manuscript as a preprint, please upload a single Preprint PDF.

Feel free to email us at plosbiology@plos.org if you have any queries relating to your submission.

Kind regards,

Richard

Richard Hodge, PhD

Senior Editor, PLOS Biology

rhodge@plos.org

PLOS

Empowering researchers to transform science

Carlyle House, Carlyle Road, Cambridge, CB4 3DN, United Kingdom

California (U.S.) corporation #C2354500, based in San Francisco

# Peer review: Decision Letter 1

**DOI:** 10.1371/journal.pbio.3003996.r002

**Authors:** Richard Hodge

24 Apr 2026

Dear Dr Xiong,

Thank you for your patience while your manuscript "Structural determinants of IGHV1-69 public antibodies conferring resilience to SARS-CoV-2 antigenic escape" was peer-reviewed at PLOS Biology. Please accept my apologies for the delays that you have experienced during the peer review process. Your manuscript has now been evaluated by the PLOS Biology editors, an Academic Editor with relevant expertise, and by three independent reviewers.

In light of the reviews, which you will find at the end of this email, we would like to invite you to revise the work to thoroughly address the reviewers' reports.

As you will see below, the reviewers agree that the structural and antibody-engineering parts of the manuscript are well done and Reviewer’s #1 and #3 provide several experimental requests to improve the structural basis and functional validation of the findings. However, Reviewer #2 focuses his/her review on the AI model aspects and raises several technical concerns with the overall strength of the antibody discovery pipeline. This includes the description of the model training data and the lack of further model evaluation such as train/validation/test split or performance metrics. In addition, the reviewer notes that negative-control validation is not included, for example with experiments selecting low-ranked or random candidates to test whether the model enriches for hits.

Given the extent of revision needed, we cannot make a decision about publication until we have seen the revised manuscript and your response to the reviewers' comments. Your revised manuscript is likely to be sent for further evaluation by all or a subset of the reviewers.

In addition to these revisions, you will need to complete some formatting changes, which you will receive in a follow up email. A member of our team will be in touch with a set of requests shortly.

We expect to receive your revised manuscript within 3 months. Please email us (plosbiology@plos.org) if you have any questions or concerns, or would like to request an extension.

At this stage, your manuscript remains formally under active consideration at our journal; please notify us by email if you do not intend to submit a revision so that we may withdraw it.

**IMPORTANT - SUBMITTING YOUR REVISION**

Your revisions should address the specific points made by each reviewer. Please submit the following files along with your revised manuscript:

1. A 'Response to Reviewers' file - this should detail your responses to the editorial requests, present a point-by-point response to all of the reviewers' comments, and indicate the changes made to the manuscript.

*NOTE: In your point-by-point response to the reviewers, please provide the full context of each review. Do not selectively quote paragraphs or sentences to reply to. The entire set of reviewer comments should be present in full and each specific point should be responded to individually, point by point.

You should also cite any additional relevant literature that has been published since the original submission and mention any additional citations in your response.

2. In addition to a clean copy of the manuscript, please also upload a 'track-changes' version of your manuscript that specifies the edits made. This should be uploaded as a "Revised Article with Changes Highlighted" file type.

*Re-submission Checklist*

When you are ready to resubmit your revised manuscript, please refer to this re-submission checklist: https://plos.io/Biology_Checklist

To submit a revised version of your manuscript, please go to https://www.editorialmanager.com/pbiology/ and log in as an Author. Click the link labelled 'Submissions Needing Revision' where you will find your submission record.

Please make sure to read the following important policies and guidelines while preparing your revision:

*Published Peer Review*

Please note while forming your response, if your article is accepted, you may have the opportunity to make the peer review history publicly available. The record will include editor decision letters (with reviews) and your responses to reviewer comments. If eligible, we will contact you to opt in or out. Please see here for more details:

https://blogs.plos.org/plos/2019/05/plos-journals-now-open-for-published-peer-review/

*PLOS Data Policy*

Please note that as a condition of publication PLOS' data policy (http://journals.plos.org/plosbiology/s/data-availability) requires that you make available all data used to draw the conclusions arrived at in your manuscript. If you have not already done so, you must include any data used in your manuscript either in appropriate repositories, within the body of the manuscript, or as supporting information (N.B. this includes any numerical values that were used to generate graphs, histograms etc.). For an example see here: http://www.plosbiology.org/article/info%3Adoi%2F10.1371%2Fjournal.pbio.1001908#s5

*Blot and Gel Data Policy*

We require the original, uncropped and minimally adjusted images supporting all blot and gel results reported in an article's figures or Supporting Information files. We will require these files before a manuscript can be accepted so please prepare them now, if you have not already uploaded them. Please carefully read our guidelines for how to prepare and upload this data: https://journals.plos.org/plosbiology/s/figures#loc-blot-and-gel-reporting-requirements

*Protocols deposition*

To enhance the reproducibility of your results, we recommend that if applicable you deposit your laboratory protocols in protocols.io, where a protocol can be assigned its own identifier (DOI) such that it can be cited independently in the future. Additionally, PLOS ONE offers an option for publishing peer-reviewed Lab Protocol articles, which describe protocols hosted on protocols.io. Read more information on sharing protocols at https://plos.org/protocols?utm_medium=editorial-email&utm_source=authorletters&utm_campaign=protocols

Thank you again for your submission to our journal. We hope that our editorial process has been constructive thus far, and we welcome your feedback at any time. Please don't hesitate to contact us if you have any questions or comments.

Best regards,

Richard

Richard Hodge, PhD

Senior Editor, PLOS Biology

rhodge@plos.org

------------------------------------

REVIEWS:

Reviewer #1: This study is comprehensive and content-rich, systematically elucidating how IGHV1-69 public antibodies acquire tolerance to SARS-CoV-2 antigenic drift through somatic hypermutation (SHM), and identifying the ultrapotent broadly neutralizing antibody ZL525 through AI-based discovery. However, further adjustments and improvements are necessary regarding the structural basis, functional validation, and reproducibility of the AI methodology.

1. Antibodies C092, C807, BD56-104, and BD56-597 acquire tolerance to the L452R and F490S mutations through SHM; however, they show varying degrees of sensitivity to the R346T mutation such as BQ.1.1. Is there structural evidence suggesting that the interaction modes of HCDR3 or LFR3 with R346 differ among these antibodies?

2. The mutant R1-32-AAM was engineered by introducing seven SHM sites into R1-32, yet it still lacks neutralizing activity against variants harboring the N354 glycosylation, such as KP.3. Does this imply that these SHM sites are insufficient to overcome the glycan barrier? Does ZL525 possess unique mutations beyond these seven sites?

3. In Fig 1 and Fig S4, R1-32 remained effective in the conformational change inhibition assay but completely lost its neutralizing activity. The authors should provide further clarification on this discrepancy.

4. Fig 3 shows that the LFR3 and LCDR3 regions of C092 are involved in RBD binding, whereas the other three antibodies do not exhibit similar characteristics. Can amino acid substitution experiments be conducted to validate the contribution of these LC regions to breadth?

5. Did the AI model account for non-affinity factors, such as glycan shielding? Is ZL525's activity against KP.3 a serendipitous discovery?

6. Does the glycan tolerance of ZL525 originate from the conformational plasticity of HCDR2? It may be beneficial to graft HCDR2 of ZL525 onto the frameworks of the four antibodies, such as C092, to verify whether this confers glycan tolerance.

Reviewer #2: Here is an assessment of the Niu et al manuscript, focusing primarily on evaluation of the AI model aspects:

The model is described as a sequence-embedding-based affinity predictor. Independent RoFormer language models are used for antibody heavy and light chain embeddings, ESM2 is used for antigen embeddings, and an MLP is fit on top of the embeddings to predict antibody-antigen binding affinity.

One challenge is that the description of the model training data is inconsistent and insufficiently specific to allow for close review. The abstract says the model was trained on integrated structural, neutralization, and binding data, whereas the Methods section says it was fine-tuned on a consolidated affinity dataset assembled from multiple public sources. Those descriptions are not equivalent, and leave it unclear what training data was actually used for which model components.

The manuscript itself does not adequately report model evaluation. The authors don't present a train/validation/test split, a data-leak avoidance strategy (e.g. a sequence-similarity-based exclusion criterion across train/validation/test splits), an external validation set, calibration analysis, or performance metrics demonstrating the model predicts affinity well.

The manuscript says that top-scoring antibodies were prioritized for experimental validation, but it is not clear how many ranked candidates were tested. The paper mainly showcases a single successful lead, ZL525, which was predicted to have higher affinity and was then experimentally validated.

Most importantly, there is no negative-control validation, for example with experiments selecting low-ranked or random candidates to test whether the model actually enriched for hits. Without that, and especially combined with the lack of reporting on model evaluation/performance, the manuscript does not provide evidence to demonstrate the value is added by the affinity prediction model.

In its current form, the structural and antibody-engineering parts of the manuscript are strong but the ML elements are weak and will need convincing dataset composition, split strategy, held-out-set performance, number of experimentally tested ranked candidates, and, if possible, prospective validation against negatives or a null model.

Reviewer #3: In this study, Niu et al. characterized how a class of IGHV1-69 public antibodies targeting SARS-CoV-2 RBD overcomes escape mutations at L452 and F490 that are found in multiple SARS-CoV-2 variants via the acquisition of somatic hypermutations. Key somatic hypermutations were identified through an in-depth characterization of four members in this public antibody class with broad reactivity (C092, C807, BD56-104, and BD56-597). These somatic hypermutations also improve cross-reactivity breadth against other SARSr-CoV strains. These results provide a mechanistic understanding of how affinity maturation drives the development of cross-reactivity breadth for this class of public antibodies. At the end, an AI model was trained to identify the antibody ZL525, which belongs to the same public antibody class as C092, C807, BD56-104, and BD56-597, but with even broader cross-reactivity. Overall, this study is well-executed and provides important insights into the interplay between SARS-CoV-2 antibody responses and viral evolution. However, there are several concerns that need to be addressed.

Major comments:

1.According to lines 150-151, Beta has no mutations at positions 452 and 490. However, the binding and neutralization of germline-like R1-32 against Beta is much weaker than against the WT (Figure 1c, e, f, and g). Is there a structural explanation?

2.The observation described in lines 367-368: "combining HCDR1 germline reversion with the HCDR3 Y109AH substitution in C092, C807, and BD56-597 produced an additive reduction in binding" contradicts with the claim in lines 369-370: "distinct antigen-contact regions act synergistically to achieve high-affinity binding." By definition, additive means no synergy.

3.Lines 535-540: "In contrast, the AI-identified antibody ZL525 exhibits greater tolerance to this glycan-mediated shielding, likely due to structural adaptations introduced by somatic hypermutation… and highlight ZL525 as a broadly neutralizing antibody capable of overcoming this glycan-mediated escape mechanism." This is a very interesting point. To increase impact of this study, the authors should elaborate on the proposed mechanism by analyzing how N354 glycan would affect the binding of C092, C807, BD56-104, and BD56-597, but not ZL525.

4.Lines 595-598: "We have shown that the synergistic effect of sequence variation and glycan-mediated structural occlusion enables viral escape from previously identified mutation-tolerant R1-32-like antibodies (C092, C807, BD56-104, and BD56-597)." This claim of synergistic effect is not supported by any data in this study.

5.Lines 616-618: "This approach is particularly powerful for targeting rapidly evolving pathogens, like SARS-CoV-2, where conventional antibody discovery methods struggle to keep pace with viral escape variants" This claim needs to be toned down. Since the model requires training data from antibodies discovered via conventional methods against antigenic variants (lines 911-914), it remains inherently limited by the same constraints as conventional antibody discovery methods.

Minor comments:

1.Lines 85-86: "We have also previously identified a class of R1-32-like public antibodies elicited in more than 50% of COVID-19 convalescents early in the pandemic." A reference is needed.

2.Lines 96-98: "Intriguingly, we found that several members of the R1-32-like public antibody class, including C092, C807, BD56-104, and BD56-597, retain robust binding to S-proteins of viral variants carrying mutations at positions 452SARS2 and 490SARS2." A reference is needed.

3.Line 115: should "IGHV1-69 encoded" be "IGHV1-69/IGLV1-40 encoded" according to line 87? Relatedly, since this study identified convergent somatic hypermutations in the light chain, it does not make sense if those antibodies are encoded by different light chain V genes.

4.It will be helpful to include a table in the supplemental information to summarize the amino acid sequences at position 452 and 490 across different SARS-CoV-2 variants.

5.Lines 186-187: "IC50 >10 µg/mL" Should that be ">100 µg/mL" according to Figure 1g?

6.Figure 2: please clarify the meaning of "S-R/6P" in the figure legend.

7.Figure S4: for some of the panels, the label at the top of the figures do not align with the corresponding lane in the gel image.

8.Lines 487-488: "based on a consolidated antigen binding affinity dataset." What dataset is that? If it is from a previous study, please provide the reference.

9.Line 533: "All four antibodies". Please indicate which four antibodies to avoid confusion.

# Author comment: Author response to Decision Letter 2

**DOI:** 10.1371/journal.pbio.3003996.r003

3 Aug 2026

Attachment Submitted filename: Response to reviewers-20260802.docx

# Peer review: Decision Letter 2

**DOI:** 10.1371/journal.pbio.3003996.r004

**Authors:** Richard Hodge

17 Aug 2026

Dear Xiaoli,

Thank you for your patience while we considered your revised manuscript "Structural determinants of IGHV1-69 public antibodies conferring resilience to SARS-CoV-2 antigenic escape" for publication as a Research Article at PLOS Biology. This revised version of your manuscript has been evaluated by the PLOS Biology editors, the Academic Editor and the original reviewers.

Based on the reviews, we are likely to accept this manuscript for publication, provided you satisfactorily address the following data and other policy-related requests that I have provided below (A-H):

(A) We routinely suggest changes to titles to ensure maximum accessibility for a broad, non-specialist readership. In this case, we would suggest the following edit to title. Please ensure you change both the manuscript file and the online submission system, as they need to match for final acceptance:

“R1-32-like public antibodies acquire tolerance to SARS-CoV-2 antigenic drift through somatic hypermutation”

(B) You may be aware of the PLOS Data Policy, which requires that all data be made available without restriction: http://journals.plos.org/plosbiology/s/data-availability. For more information, please also see this editorial: http://dx.doi.org/10.1371/journal.pbio.1001797

Note that we do not require all raw data. Rather, we ask that all individual quantitative observations that underlie the data summarized in the figures and results of your paper be made available in one of the following forms:

-Supplementary files (e.g., excel). Please ensure that all data files are uploaded as 'Supporting Information' and are invariably referred to (in the manuscript, figure legends, and the Description field when uploading your files) using the following format verbatim: S1 Data, S2 Data, etc. Multiple panels of a single or even several figures can be included as multiple sheets in one excel file that is saved using exactly the following convention: S1_Data.xlsx (using an underscore).

-Deposition in a publicly available repository. Please also provide the accession code or a reviewer link so that we may view your data before publication.

Regardless of the method selected, please ensure that you provide the individual numerical values that underlie the summary data displayed in the following figure panels as they are essential for readers to assess your analysis and to reproduce it:

Figure 1D-G, 4B-G, 6B-C, S1A-B, S2, S3A-D, S8B-C, S8E, S8H, S8K-L, S11A-D, S12A-F, S13A-C, S16A-B, S16D, S17C-D, S18C-D

NOTE: the numerical data provided should include all replicates AND the way in which the plotted mean and errors were derived (it should not present only the mean/average values).

(C) Please also ensure that each of the relevant figure legends in your manuscript include information on *WHERE THE UNDERLYING DATA CAN BE FOUND*, and ensure your supplemental data file/s has a legend.

(D) Thank you for providing the raw and uncropped gel images for the following figures:

Figure S4A-F

At this stage, we ask that the raw images are prepared in a separate PDF file (named S1_raw_images) in the Supplementary Information.

(E) Please ensure that your Data Statement in the submission system accurately describes where your data can be found and is in final format, as it will be published as written there.

(F) Per journal policy, if you have generated any custom code during the course of this investigation, please make it available without restrictions. Please ensure that the code is sufficiently well documented and reusable, and that your Data Statement in the Editorial Manager submission system accurately describes where your code can be found. More information on our Code Policy, what and how to share can be found here: https://journals.plos.org/plosbiology/s/code-availability

Please note that we cannot accept sole deposition of code in GitHub, as this could be changed after publication. However, you can archive this version of your publicly available GitHub code to Zenodo. Once you do this, it will generate a DOI number, which you will need to provide in the Data Accessibility Statement (you are welcome to also provide the GitHub access information). See the process for doing this here: https://docs.github.com/en/repositories/archiving-a-github-repository/referencing-and-citing-content

(G) Please ensure that you are using best practice for statistical reporting and data presentation. These are our guidelines https://journals.plos.org/plosbiology/s/best-practices-in-research-reporting#loc-statistical-reporting and a useful resource on data presentation https://journals.plos.org/plosbiology/article?id=10.1371/journal.pbio.1002128

- If you are reporting experiments where n ≤ 5, please plot each individual data point.

(H) Please note that per journal policy, the model system/species studied should be clearly stated in the abstract of your manuscript (human).

------------------------------------------------------------------------

As you address these items, please take this last chance to review your reference list to ensure that it is complete and correct. If you have cited papers that have been retracted, please include the rationale for doing so in the manuscript text, or remove these references and replace them with relevant current references. Any changes to the reference list should be mentioned in the cover letter that accompanies your revised manuscript.

In addition to these revisions, you may need to complete some formatting changes, which you will receive in a follow up email. A member of our team will be in touch with a set of requests shortly. If you do not receive a separate email within a few days, please assume that checks have been completed, and no additional changes are required.

We expect to receive your revised manuscript within two weeks.

To submit your revision, please go to https://www.editorialmanager.com/pbiology/ and log in as an Author. Click the link labelled 'Submissions Needing Revision' to find your submission record. Your revised submission must include the following:

- a cover letter that should detail your responses to any editorial requests, if applicable, and whether changes have been made to the reference list

- a Response to Reviewers file that provides a detailed response to the reviewers' comments (if applicable, if not applicable please do not delete your existing 'Response to Reviewers' file.)

- a track-changes file indicating any changes that you have made to the manuscript.

NOTE: If Supporting Information files are included with your article, note that these are not copyedited and will be published as they are submitted. Please ensure that these files are legible and of high quality (at least 300 dpi) in an easily accessible file format. For this reason, please be aware that any references listed in an SI file will not be indexed. For more information, see our Supporting Information guidelines:

https://journals.plos.org/plosbiology/s/supporting-information

*Published Peer Review History*

Please note that you may have the opportunity to make the peer review history publicly available. The record will include editor decision letters (with reviews) and your responses to reviewer comments. If eligible, we will contact you to opt in or out. Please see here for more details:

https://plos.org/published-peer-review-history/

*Press*

Should you, your institution's press office or the journal office choose to press release your paper, please ensure you have opted out of Early Article Posting on the submission form. We ask that you notify us as soon as possible if you or your institution is planning to press release the article.

*Protocols deposition*

To enhance the reproducibility of your results, we recommend that if applicable you deposit your laboratory protocols in protocols.io, where a protocol can be assigned its own identifier (DOI) such that it can be cited independently in the future. Additionally, PLOS ONE offers an option for publishing peer-reviewed Lab Protocol articles, which describe protocols hosted on protocols.io. Read more information on sharing protocols at https://plos.org/protocols?utm_medium=editorial-email&utm_source=authorletters&utm_campaign=protocols

Please do not hesitate to contact me should you have any questions.

Best wishes,

Richard

Richard Hodge, PhD

Senior Editor, PLOS Biology

rhodge@plos.org

------------------------------------------------------------------------

Reviewer remarks:

Reviewer #1: the authors have addressed all my concerns.

Reviewer #2: The authors appear to have addressed the concerns, and I have no further comments.

Reviewer #3: The authors have addressed all my previous concerns.

# Author comment: Author response to Decision Letter 3

**DOI:** 10.1371/journal.pbio.3003996.r005

27 Aug 2026

Attachment Submitted filename: Response_to_reviewers-20260802_auresp_3.docx

# Editor report: Decision Letter 3

**DOI:** 10.1371/journal.pbio.3003996.r006

**Authors:** Richard Hodge

31 Aug 2026

Dear Xiaoli,

Thank you for the submission of your revised Research Article "R1-32-like public antibodies acquire tolerance to SARS-CoV-2 antigenic drift through somatic hypermutation" for publication in PLOS Biology. On behalf of my colleagues and the Academic Editor, Ali Ellebedy, I am pleased to say that we can in principle accept your manuscript for publication, provided you address any remaining formatting and reporting issues. These will be detailed in an email you should receive within 2-3 business days from our colleagues in the journal operations team; no action is required from you until then. Please note that we will not be able to formally accept your manuscript and schedule it for publication until you have completed any requested changes.

Please take a minute to log into Editorial Manager at http://www.editorialmanager.com/pbiology/, click the "Update My Information" link at the top of the page, and update your user information to ensure an efficient production process.

PRESS

We frequently collaborate with press offices. If your institution or institutions have a press office, please notify them about your upcoming paper at this point, to enable them to help maximise its impact. If the press office is planning to promote your findings, we would be grateful if they could coordinate with biologypress@plos.org. If you have previously opted in to the early version process, we ask that you notify us immediately of any press plans so that we may opt out on your behalf.

We also ask that you take this opportunity to read our Embargo Policy regarding the discussion, promotion and media coverage of work that is yet to be published by PLOS. As your manuscript is not yet published, it is bound by the conditions of our Embargo Policy. Please be aware that this policy is in place both to ensure that any press coverage of your article is fully substantiated and to provide a direct link between such coverage and the published work. For full details of our Embargo Policy, please visit http://www.plos.org/about/media-inquiries/embargo-policy/.

Thank you again for choosing PLOS Biology for publication and supporting Open Access publishing. We look forward to publishing your study.

Sincerely,

Melissa

Melissa Vázquez Hernández, PhD

Associate Editor

PLOS Biology

mvazquezhernandez@plos.org

on behalf of

Richard Hodge, PhD, PhD

Senior Editor

PLOS Biology

rhodge@plos.org
